# Ability of Local Clearance of Senescent Cells in Ipsilateral Hemisphere to Mitigate Acute Ischemic Brain Injury in Mice

**DOI:** 10.7150/ijbs.84060

**Published:** 2023-05-29

**Authors:** Kuan-Jung Lu, Joen-Rong Sheu, Ruei-Dun Teng, Tanasekar Jayakumar, Chi-Li Chung, Cheng-Ying Hsieh

**Affiliations:** 1 Graduate Institute of Medical Sciences, College of Medicine, Taipei Medical University, Taipei, Taiwan.; 2 Department of Pharmacology, School of Medicine, College of Medicine, Taipei Medical University, Taipei, Taiwan.; 3 Division of Pulmonary Medicine, Department of Internal Medicine, Taipei Medical University Hospital, Taipei, Taiwan.; 4 Division of Pulmonary Medicine, Department of Internal Medicine, School of Medicine, College of Medicine, Taipei Medical University, Taipei, Taiwan.; 5 Department of Ecology & Environmental Sciences, School of Life Science, Pondicherry University, Kalapet, India.; 6 School of Respiratory Therapy, College of Medicine, Taipei Medical University, Taipei, Taiwan.

**Keywords:** senolytic treatment, acute ischemic stroke, lenti-INK-ATTAC viral vector

## Abstract

Senolytic treatment has potential therapeutic efficacy for acute ischemic stroke (AIS). However, the systemic treatment of senolytics may produce off-target side effects and a toxic profile, which affect analysis of the role of acute senescence of neuronal cells in pathogenesis of AIS. We constructed a novel lenti-INK-ATTAC viral vector to introduce INK-ATTAC genes to the ipsilateral brain and locally eliminate senescent brain cells by administering AP20187 to activate caspase-8 apoptotic cascade. In this study, we have found that acute senescence is triggered by middle cerebral artery occlusion (MCAO) surgery, particularly in astrocytes and cerebral endothelial cells (CECs). The upregulation of p16^INK4a^ and senescence-associated secretory phenotype (SASP) factors including matrix metalloproteinase-3, interleukin-1 alpha and -6 were observed in oxygen-glucose deprivation-treated astrocytes and CECs. The systemic administration of a senolytic, ABT-263, prevented the impairment of brain activity from hypoxic brain injury in mice, and significantly improved the neurological severity score, rotarod performance, locomotor activity, and weight loss. The treatment of ABT-263 reduced senescence of astrocytes and CECs in MCAO mice. Furthermore, the localized removal of senescent cells in the injured brain through the stereotaxical injection of lenti-INK-ATTAC viruses generates neuroprotective effects, protecting against acute ischemic brain injury in mice. The content of SASP factors and mRNA level of p16^INK4a^ in the brain tissue of MCAO mice were significantly reduced by the infection of lenti-INK-ATTAC viruses. These results indicate that local clearance of senescent brain cells is a potential therapy on AIS, and demonstrate the correlation between neuronal senescence and pathogenesis of AIS.

## Introduction

The prevalence of stroke has increased because of the growth in the older adult population and the industrialization of countries in the Global South; this has strongly affected worldwide health expenses.^1^ The medical treatment of acute ischemic stroke (AIS) is limited to intravenous thrombolytic therapy with a recombinant tissue plasminogen activator (rt-PA); however, the length of treatment and hemorrhagic side effects often prevent the use of rt-PA in clinical settings.^2^ Therefore, innovative thrombolytics with few adverse side effects and potent neuroprotective therapies that facilitate functional recovery after acute ischemic brain injury must be developed.^3^ One therapeutic strategy for eliminating senescent cells has been reported to alleviate neurodegenerative diseases and the associated cognitive disabilities.^4^ This senolytic approach has exhibited potential therapeutic efficacy for several brain diseases.^5^, ^6^

Senescence is precarious in age-related diseases because it can influence lifespan.^7^ Senescent cells can strongly express the p16^INK4a^ and p53/p21 pathways and lead to permanent cell cycle arrest.^7^-^9^ Cellular senescence is accompanied by a special senescence-associated secretory phenotype (SASP), which synthesizes and produces proinflammatory cytokines, chemokines, growth factors, and metalloproteinases involved in inflammatory processes.^10^, ^11^ Herranz et al. indicated that the *in vivo* deleterious effect of senescent cells strongly depends on the effects of SASP on the surrounding microenvironment and the related immune responses.^12^ Senescence can be acute or chronic depending on the duration and other characteristics. Acute senescence contributes to the ordinary biological processes of embryonic development and tissue repair. Chronic senescence, which is exacerbated by consistent exposure to stress, can cause cellular and tissue damage.^13^ The senescence of neuronal cells can affect the pathophysiology of various neurodegenerative diseases such as Alzheimer's disease and Parkinson's disease.^14^ Removing senescent cells prevents gliosis, neurofibrillary tangle deposition, and the degeneration of cortical and hippocampal neurons, thereby preserving cognitive function in Alzheimer's disease.^4^ The discovery of senolytic drugs, which eliminate senescent cells, led to novel cures for age‐related disorders.^15^ However, removing senescent cells entails several challenges. For example, although senolytic drugs selectively target senescent cells, they can damage neighboring organisms.^7^ Cell clearance by these drugs also affects nonsenescent cells.^15^ Transient thrombocytopenia and neutropenia are common side effects among patients prescribed senolytic drugs.^16^ In addition, cellular senescence can facilitate tissue-wound healing in some organs.^17^ One study indicated that senolytic drugs can alleviate ischemic brain injury considerably,^6^ but whether this effect arises from specifically targeting senescent brain cells or from the systemic effects is unclear. The local elimination of senescent cells in the brain after hypoxia can either elucidate the pathological role of acute senescence in the brain after AIS or serve as a potential therapy without nonspecific senolytic side effects.

Baker et al. developed a genetic approach to eliminate senescent cells *in vivo*.^18^ They designed a transgenic p16INK-apoptosis through the targeted activation of caspase 8 (ATTAC) mice that killed senescent cells by using the dimerizing agent AP20187. AP20187 activates the FKBP-CASP8-fused protein, a chimeric protein consisting of the binding domain of the FK506-binding protein (FKBP) and the protease domain of caspase-8, which ablates *p16*^Ink4a^-positive senescent cells in INK-ATTAC transgenic mice^19^; the agent eliminated senescent cells throughout the body. We constructed a lentiviral vector based on the INK-ATTAC model to locally eliminate senescent cells in the brain. To determine the role of the acute senescence of brain cells after AIS, we identified the major senescent brain cells of AIS-induced acute senescence and examined the recovery of brain activity through senolytic therapy. In addition, we used a novel lenti-INK-ATTAC viral vector to locally remove senescent cells in the brain and the neuroprotective effects of senescent cell removal.

## Methods

This article follows the *International Journal of Biological Science* implementation of Transparency and Openness Promotion Guidelines. Detailed description of methodology is available in the Supplemental Material.

### Animals

C57BL/6 male mice (6-8 weeks old) were used. The Institutional Animal Care and Use Committee of Taipei Medical University approved the study's methods, which complied with the Guide for the Care and Use of Laboratory Animals (NIH publication no. 85-23, 1996).

### Statistics

All results are presented as means ± standard errors of the means (SEMs). All statistical analyses were performed using Prism (version 9.02, GraphPad Software, San Diego, CA, USA). Significant differences between groups were calculated using a two-tailed unpaired Student's *t* test. Groups were compared using one-way analyses of variance followed by Tukey's posttest analyses for the significance between isolated groups. *P* < 0.05 indicated statistical significance.

## Results

### Expression of acute senescence in the ipsilateral side of C57BL/6 mice after MCAO

**Figure 1:**
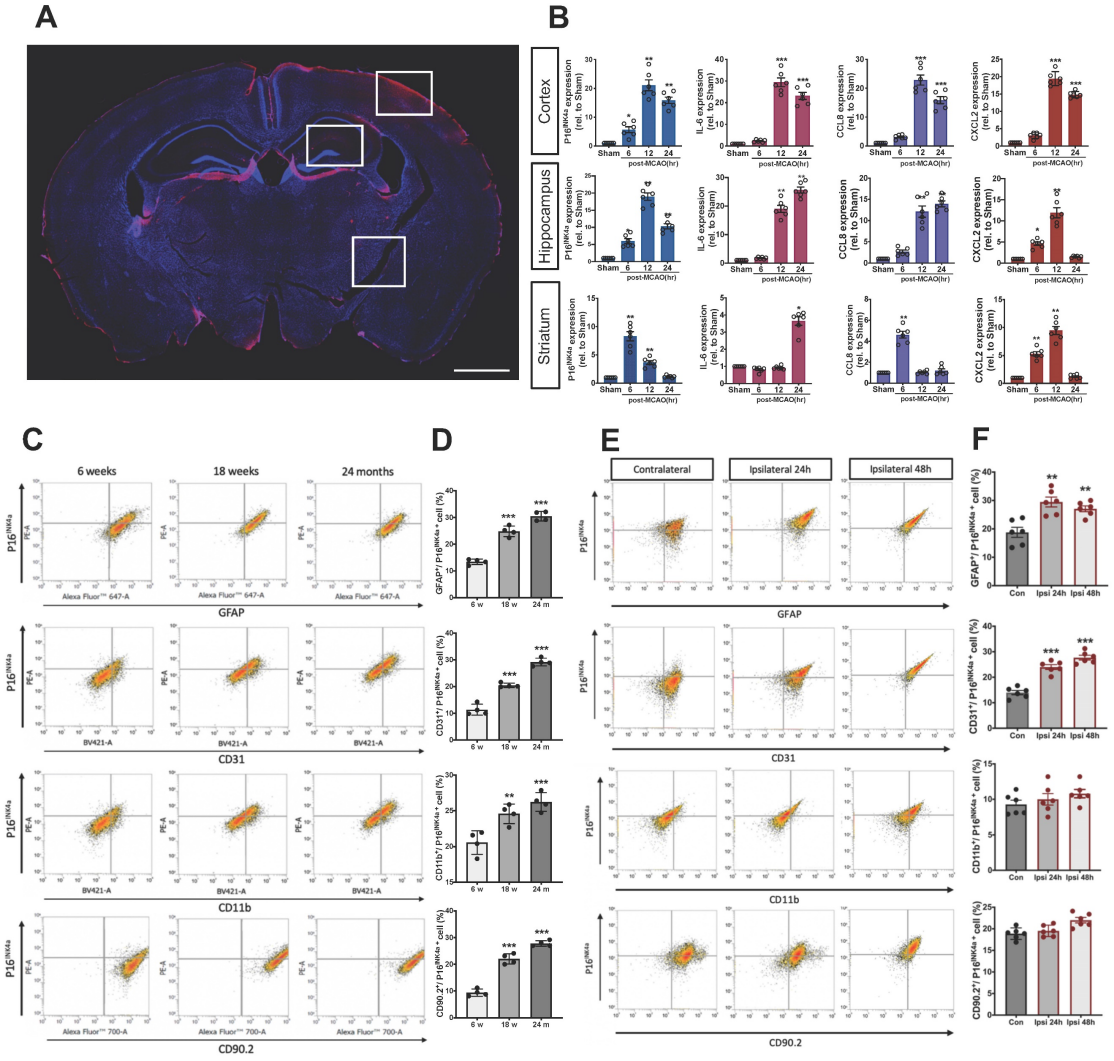
** Expression of p16^INK4a^, a typical marker of senescence, and SASP factors in the ipsilateral brain of C57BL/6 mice after MCAO. (A)** A representative photo of the immunohistochemistry staining of p16^INK4a^ in the ipsilateral (Ipsi) brain of mice subjected to MCAO taken using the EVOS FL Cell Imaging System. The photograph represents three similar experiments. Blue depicts the nucleus, red depicts p16^INK4a^, and the white bar represents 1 mm. **(B)** The mRNA levels of cellular senescence markers, namely p16^INK4a^, IL-6, CCL8, and CXCL2, were determined in the cortex, hippocampus, and striatum of the mice 6, 12, and 24 h after MCAO. All data are represented as means ± SEMs (n = 8); ^*^P < 0.05, ^**^P < 0.01, and ^***^P < 0.001, compared with sham control group. The percentage change in senescent neuronal cells, namely cerebral endothelial cells, astrocytes, microglia, and neurons **(C)** in the brain of 6 weeks, 18 weeks and 24 months old mice or **(E)** in the ipsilateral brain 24 h and 48 h after MCAO was analyzed using flow cytometry. **(D&F)** Quantification of the flow cytometry analysis. All data are represented as means ± SEMs (n = 4 (C); n = 6 (E)). ^**^P < 0.01, and ^***^P < 0.001, compared with the 6-weeks group (C) or contralateral (Con) group (E).

P16^INK4a^, a typical marker of cellular senescence,^20^ was observed in the ipsilateral cortex, hippocampus, and striatum 24 h post-MCAO in the mice by using immunohistochemistry staining (Fig. 1A). The mRNA levels of senescence markers, namely p16^INK4a^, IL-6, CCL8, and CXCL2^10^, ^21^ in the ipsilateral side of the cortex, hippocampus, and striatum, were evaluated using a real-time PCR at three time points after MCAO. The mRNA levels of p16^INK4a^, IL-6, CCL8, and CXCL2 in the ipsilateral cortex increased at 6 h and demonstrated substantial elevation at 12 and 24 h after MCAO (Fig. 1B). A significant upregulation of p16^INK4a^, IL-6, CCL8, and CXCL2 was also observed 12 h after MCAO in the ipsilateral side of the hippocampus, but CXCL2 decreased 24 h post-MCAO. In the ipsilateral striatum, senescence marker p16^INK4a^ increased at 6 h before decreasing at 12 and 24 h post-MCAO. Regarding inflammatory cytokines, the IL-6 level increased at 24 h post-MCAO, and chemokines CCL8 and CXCL2 increased at 6 h post-MCAO and then decreased (Fig. 1B). These results indicate that substantial senescence occurred on the ipsilateral side of ischemic stroke mice after 12 h. Thus, acute senescence occurs in the pathophysiology of ischemic stroke.

Additionally, we used a flowcytometric technique to determine the p16^INK4a^ expression of four cell types of neuronal cells in aged mice or mice with MCAO surgery. Cell surface markers, namely astrocyte marker (GFAP), cerebral endothelial cells (CECs) marker (CD31), microglia marker (CD11b), neuron marker (CD90.2), and were used to identify different neuronal cells in flowcytometric analysis. As shown in Figs. 1C and D, when compared to young mice, the proportion of senescent, astrocytes, CECs, microglia and neurons was significantly increased in 24 months-old mice compared to 6 weeks-old mice, GFAP^+^/p16^INK4a+^ (13.4 % ± 1.1 % to 30.5 % ± 1.8 %), CD31^+^/p16^INK4a+^ (11.4 % ± 2.1 % to 29.2 % ± 1.4 %), CD11b^ +^/p16^INK4a+^ (20.6 % ± 1.7 % to 26.6 % ± 1.3 %), and CD90.2^+^/p16^INK4a+^ cells (9.4 % ± 1.3 % to 27.8 % ± 1.0 % (*n* = 4, *P* < 0.001)). On the other hand, the GFAP^+^/p16^INK4a+^ and CD31^+^/p16^INK4a+^ cells significantly increased 24 h after MCAO, from 13.9 % ± 0.8 % to 24.1 % ± 0.9 % (*n* = 6, *P* < 0.01) and from 18.8 % ± 1.8 % to 24.5 % ± 1.7 % (*n* = 6, *P* < 0.001), respectively. However, no significant differences were observed in the CD11b^ +^/p16^INK4a+^ and CD90.2^+^/p16^INK4a+^ cells. Moreover, we also performed the flowcytometric analysis in mice 48 h after MCAO to clarify the time tendency, the pattern of the AIS-triggered neural cell senescence remains similar to 24 h-groups (Figs. 1E and F).

### Hypoxia triggers cellular senescence in astrocytes and cerebral endothelial cells *in vivo* and *in vitro*

**Figure 2:**
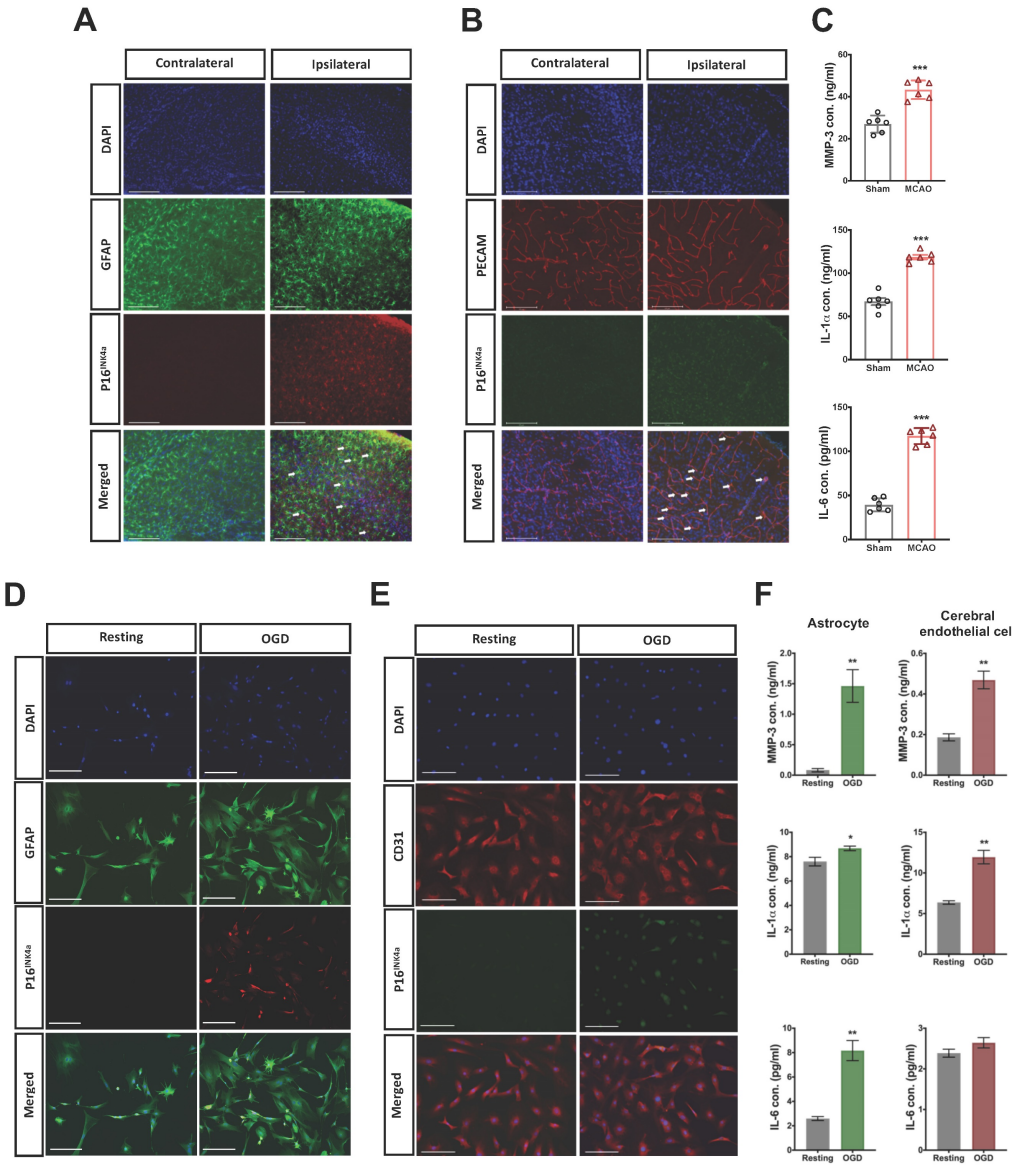
** Acute cellular senescence was provoked in astrocytes and CECs of ipsilateral cortex through MCAO and in OGD-stimulated isolated primary astrocytes and CECs.** The expression of senescence marker p16^INK4a^ in **(A)** astrocytes and **(B)** CECs was observed using immunohistochemistry staining and confocal microscopy in the ipsilateral cortex of mice 24 h after MCAO. Arrows indicate senescent cells, and white bars represent 125 μm. **(C)** The content of SASP factors, namely MMP-3, IL-6, and IL-1α, in the brain tissue of mice without or with MCAO surgery was evaluated using ELISA kits. Data are presented as means ± SEMs (*n* = 4-6); ^***^P < 0.001, compared with the sham control group. The expression of p16^INK4a^ in the isolated primary astrocytes **(D)** and CECs **(E)** was triggered by 4.5-h OGD and determined after 24-h by using confocal microscopy. The white bars represent 50 μm. **(F)** The concentration of MMP-3, IL-1α, and IL-6 in the conditioned medium of primary astrocytes and CECs stimulated by OGD was determined using ELISA kits. Data are presented as means ± SEMs (*n* = 3); ^*^P < 0.05 and ^**^P < 0.01, compared with the resting group.

The expression of cellular senescence in astrocytes and CECs after acute hypoxic brain injury was further identified through the counterstaining of p16^INK4a^ and GFAP or PECAM (CEC marker) in the injured brain. The p16^INK4a^ positive astrocytes and CECs were highly expressed in the infarct area 24 h after MCAO (Fig. 2 A and B). However, p16^INK4a^ was scant in the contralateral brain. We also determined the content of typical SASP factors, namely MMP-3, IL-1α, and IL-6 21, in the injured brain 24 h post-MCAO by using ELISA kits, and the SASP factors in brain were significantly induced by MCAO (Fig. 2C).

Primary astrocytes and CECs were harvested from the fresh brain tissue of the mice,^22^, ^23^ which were subjected to oxygen-glucose deprivation/reperfusion (OGD/R). The astrocytes and CECs were treated with 4.5 h of OGD and followed by 24 h of reperfusion. Substantial expression of p16^INK4a^ was induced after 4.5 h of OGD/R in both astrocytes and CECs (Fig. 2D and E). We then collected the conditioned medium of OGD/R-stimulated astrocytes and CECs for the ELISA assay of SASP factors. The expression of MMP-3, IL-1α, and IL-6 was significantly triggered in astrocytes by OGD/R, and the content of MMP-3 and IL-1α increased in the conditioned medium of OGD/R-stimulated CECs, but IL-6 did not (Fig. 2F).

### Ability of posttreatment of senolytic drug ABT-263 to alleviate MCAO-induced brain injury in C57BL/6 mice

**Figure 3:**
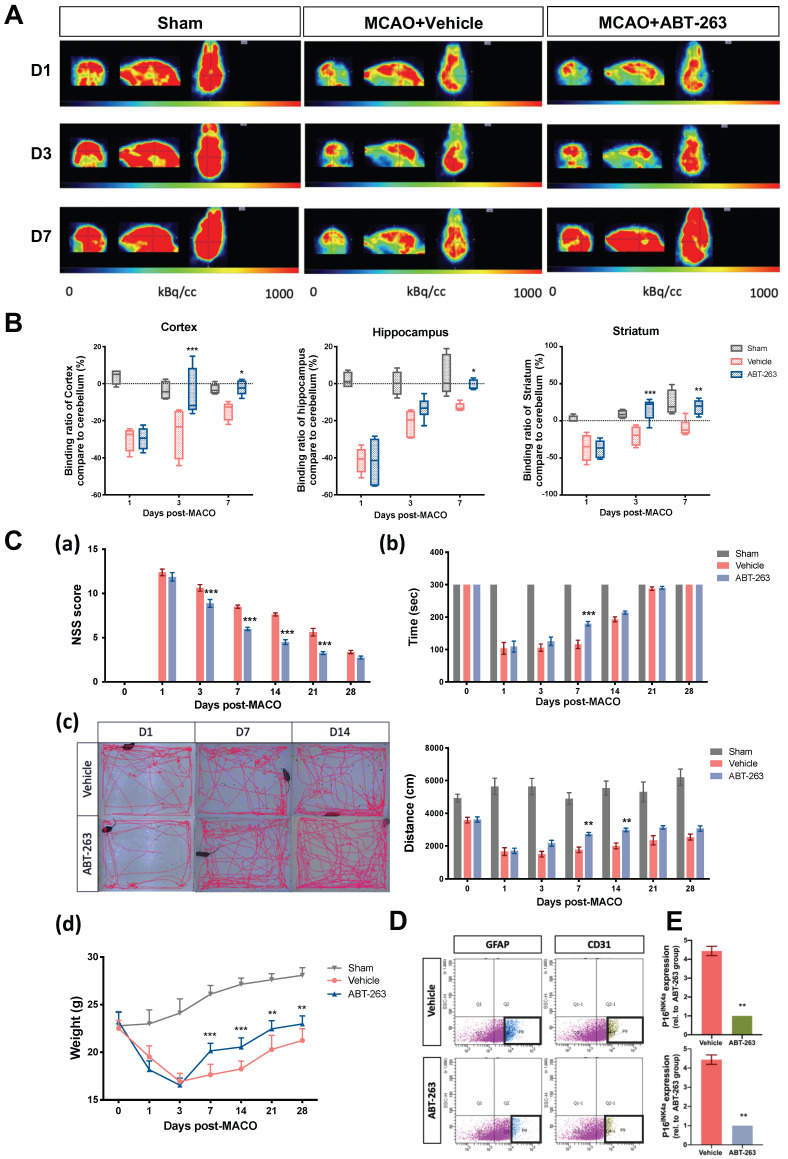
** Oral administration of senolytic ABT-263 alleviates the impairment of brain activity and neurological deficits through MCAO in C57BL/6 mice. (A)** 18F-FDG PET of the ipsilateral cortex, hippocampus, and striatum in mice subjected to MCAO was performed on Days 1, 3, and 7 after surgery. Mice were treated with or without ABT-263. **(B)** The relative 18F-FDG binding ratio of the ipsilateral cortex, hippocampus, and striatum to the cerebrum in the mice treated with or without ABT-263 on Days 1, 3, and 7 post-MCAO. **(C)** The NSSs **(a)**, rotarod test **(b)**, locomotor activity **(c)**, and body weight change **(d)** of the mice treated with or without ABT-263 were examined 0, 1, 3, 7, 14, 21, 28 days after MCAO. **(D)** Astrocyte (cell marker: GFAP) and CECs (cell marker: CD31) of mice treated with or without ABT-263 7 days after MCAO were sorted by FACS. **(E)** The mRNA levels of astrocytes and CECs in the mice treated with or without ABT-263. The mRNA expression of each gene was normalized using GAPDH mRNA expression. Data are presented as means ± SEMs (*n* = 8) ^*^P < 0.05, ^**^P < 0.01, and ^***^P < 0.001, compared with the vehicle group.

Targeting Bcl-xl, a member of the antiapoptotic Bcl-2 family, has been reported to reduce the viability of senescent cells but not that of nonsenescent cells.^24^ ABT-263 (navitoclax), which targets several Bcl-2 family proteins, including Bcl-xl, Bcl-2, and Bcl-w, is senolytic in human endothelial cells and IMR90 cells.^25^ For treating neurodegenerative diseases, ABT-263 can eliminate senescent glial cells to prevent tau-dependent pathologies and cognitive decline.^4^ We used ABT-263 to target senescent cells and identified a therapeutic effect against ischemic stroke-induced brain injury. After MCAO surgery, however, it is known that cerebral ischemia can activate two general pathways of apoptosis during the early stage. 26 To prevent the aggravation of apoptotic stress in the early stage of ischemic stroke pathogenesis, ABT-263 was administrated orally 24 h post-MCAO in the mice with ischemic stroke. Preclinical studies found out that the demand and use of glucose revealed the brain activity of the mice.^27^ Then, 18F-fluorodeoxyglucose (18F-FDG) PET was used to monitor brain activity in the mice subjected to MCAO. The oral treatment of ABT-263 (50 mg/kg/day) for 5 consecutive days significantly restored glucose demand and use in the ipsilateral cortex, hippocampus, and striatum of mice (Fig. 3A and B). NSSs, locomotor activity, rotarod performance, and weight loss were improved by the administration of ABT-263 (Fig. 3C). In addition to evaluating the therapeutic effects of ABT-263 on brain activity and neurological deficits, we further determined the eliminating effects of ABT-263 on senescent astrocytes and CECs in mice subjected to MCAO. The fluorescence-activated cell sorting (FACS) method was used to collect astrocytes and CECs in the injured brain of mice administered with or without senolytic, ABT-263. The treatment of ABT-263 substantially decreased the mRNA level of p16^INK4a^ in astrocytes and CECs at 7 days after MCAO surgery in mice (Figs. 3D and E).

### Assessment of efficacy of lenti-INK-ATTAC viral vector in H_2_O_2_-treated HEK293 cells and C57BL/6 mice with MCAO

**Figure 4:**
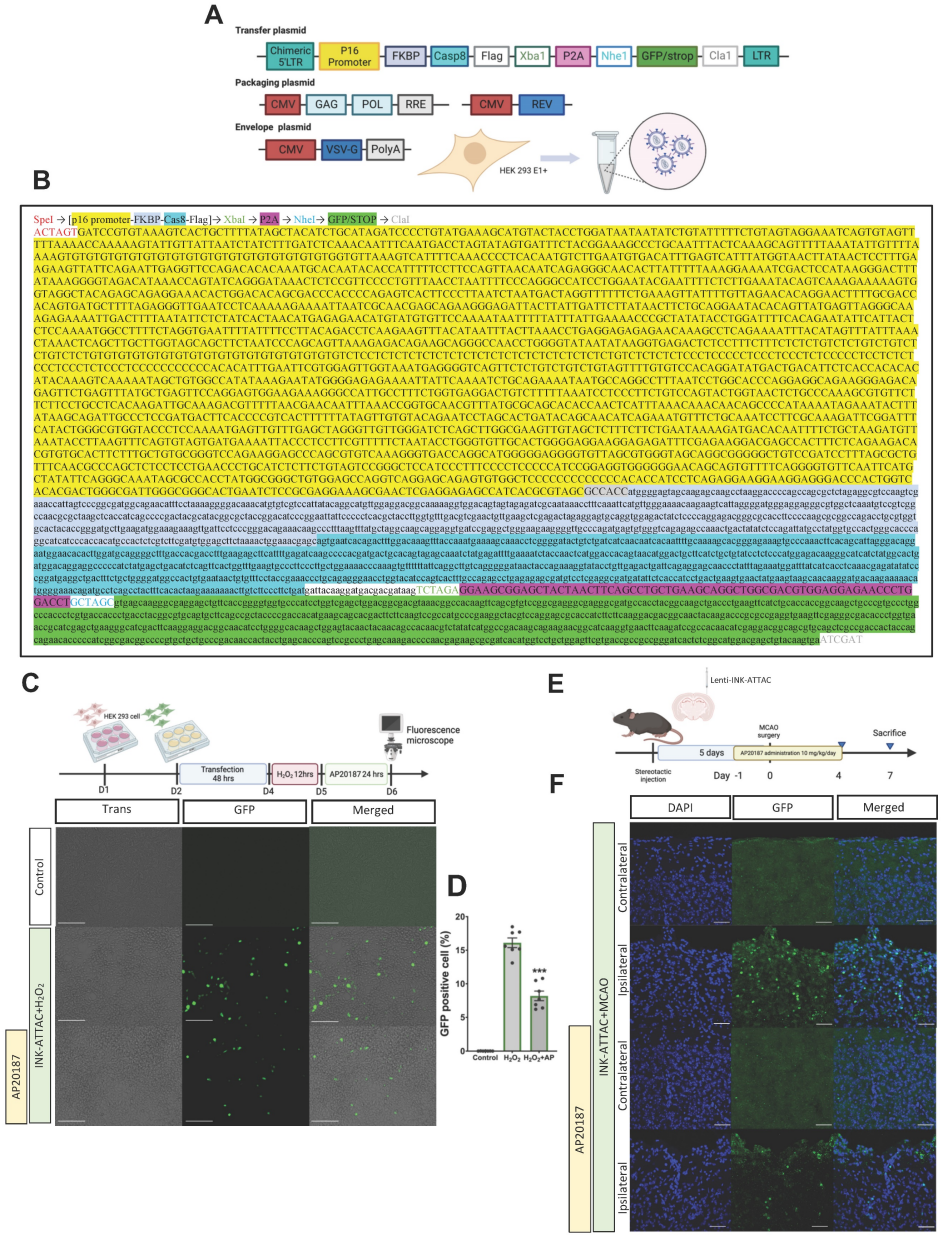
** Elimination of senescent cells through infection of lenti-INK-ATTAC viruses in H_2_O_2_-treated HEK293 cells and C57BL/6 mice subjected to MCAO. (A)** The third-generation lenti-viral vector was used to label p16^INK4a^-expressing cells. LTR: long terminal repeats, CMV: cytomegalovirus. **(B)** Full sequence of the novel lent-INK-ATTAC viral vector. **(C)** *In vitro* experiment. HEK293 cells were transfected with lenti-INK-ATTAC viruses for 48 h and then stimulated with or without H_2_O_2_ (150 μM) for 4 h. H_2_O_2_ -treated cells were then treated with or without AP20187 for 24 h. **(D)** The GFP-positive cells were observed using confocal microscopy. The white bar represents 75 μm. **(E)** The infection of lenti-INK-ATTAC viruses was performed 5 days before MCAO and AP20187 was treated 1 day before MCAO for 5 consecutive days.** (F)** Immunohistochemistry staining was used to determine the expression of GFPs in lenti-INK-ATTAC-infected C57BL/6 mice with MCAO and with or without AP20187 treatment. Blue depicts the nucleus, green depicts GFPs, and the white bar represents 50 μm. Data are presented as means ± SEMs (*n* = 5); ^***^P < 0.001, compared with the H_2_O_2_ control group.

Although the oral administration of ABT-263 considerably alleviated ischemic brain injury, this effect may not have originated from targeting senescence specifically in the brain or from the generalized effects produced by the drug. Additionally, oral administration of senolytic drugs may cause systemic off-target side effects and a toxic profile.^28^ We thus designed a novel lenti-INK-ATTAC viral vector to specifically eliminate senescent cells in the brain tissue (Fig. 4A-B). To determine the efficiency of this vector in eliminating senescent cells, HEK293 cells were infected with lenti-INK-ATTAC viruses for 48 h. The infected cells were stimulated by H_2_O_2_ (150 μM) for 12 h to trigger cellular senescence^29^ and were then treated with or without AP20187 (100 nM) for 24 h (Fig. 4C). H_2_O_2_ significantly increased the expression of GFP-positive cells (senescent cells) within lenti-INK-ATTAC-infected HEK293 cells, and the administration of AP20187 substantially eliminated GFP-positive cells (from 16.2 % ± 0.5 % to 8.3 % ± 0.5 %; *n* = 5, *P* < 0.001; Fig. 4D). We also determined the efficiency of the lenti-INK-ATTAC viral vector in an *in vivo* mouse model of MCAO. The concentrated lentiviruses were stereotaxically injected at four sites in the cortex and hippocampus of the mice brains 5 days before MCAO, which were then treated with or without AP20187 (10 mg/kg/day) for 5 consecutive days from the day before MCAO (Fig. 4E). The GFP-positive cells significantly increased in the ipsilateral brain after MCAO, and the treatment of AP20187 substantially attenuated the expression of GFP-positive cells 7 days post-MCAO (Fig. 4F). Therefore, the novel lenti-INK-ATTAC viral vector can effectively remove p16^Ink4a^-expressing cells both *in vitro* and* in vivo*.

### Localized removal of senescent cells in brain through stereotaxical injection of lenti-INK-ATTAC viral vector

**Figure 5:**
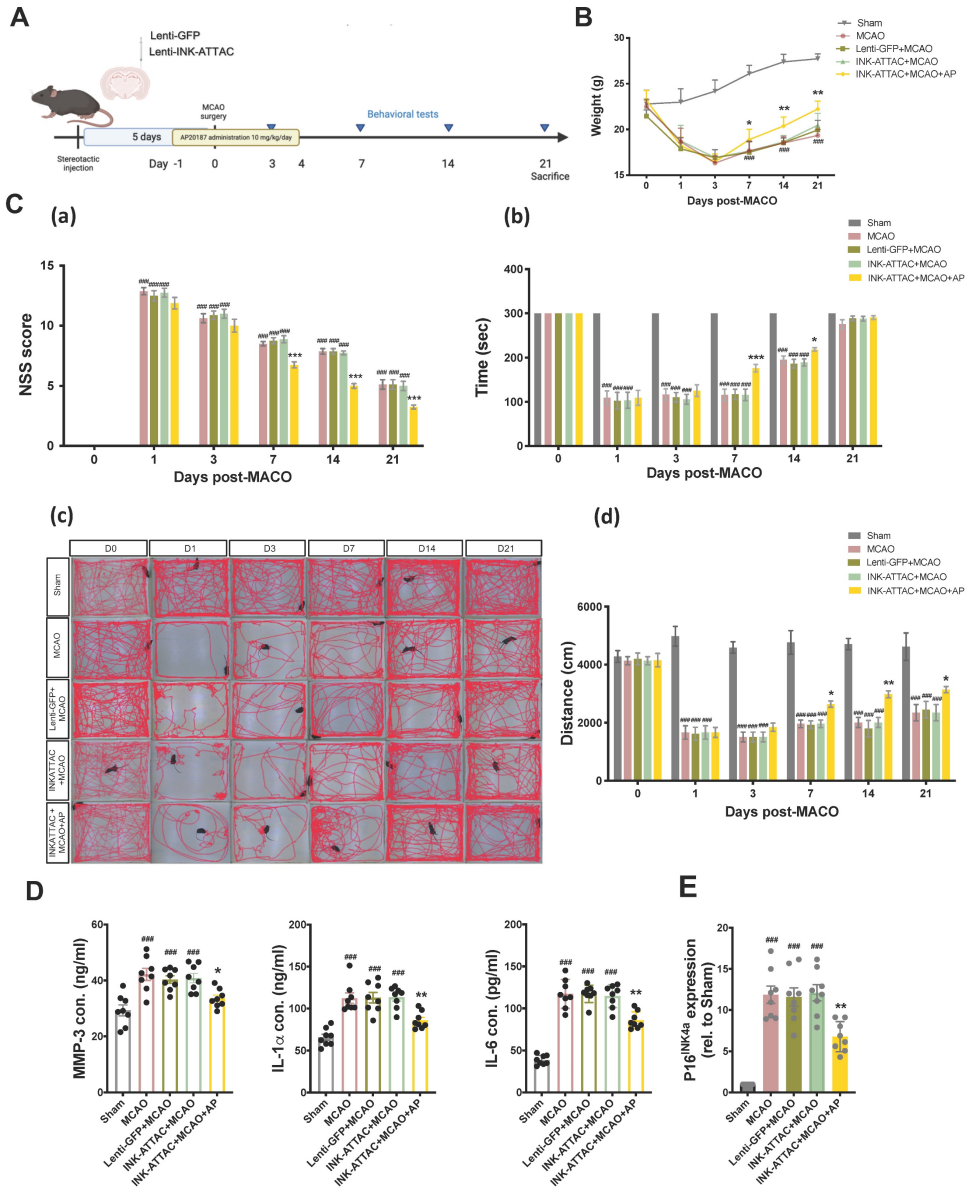
** Neuroprotective effects of local elimination of senescent cells through stereotaxical injection of lenti-INK-ATTAC viruses in the ipsilateral brain of C57BL/6 mice after acute hypoxic brain injury. (A)** Lenti-GFP or Lenti-INK-ATTAC virus injection, AP20187 administration, and data collection. **(B)** Body weight change **(C)** The NSSs **(a)**, rotarod test **(b)**, locomotor activity **(c and d)** of mice infected with or without lenti-GFP or lenti-INK-ATTAC and treated with or without AP20187 (AP) were examined 0, 1, 3, 7, 14, 21 days after MCAO. Data are presented as means ± SEMs (*n* = 12); ^###^*P* < 0.001, compared with the sham group. ^*^P < 0.05 and ^***^P < 0.001, compared with the INK-ATTAC+MCAO group.** (C and D)** The concentration of MMP-3, IL-1α, and IL-6, and the mRNA expression of p16^INK4a^ in the brain tissue of MCAO mice infected with or without lenti-GFP or lenti-INK-ATTAC and treated with or without AP20187 were evaluated using ELISA kits and quantitative reverse transcription PCR, respectively. Data are presented as means ± SEMs (*n* = 8); ^###^*P* < 0.001, compared with the sham group. ^*^P < 0.05 and ^**^P < 0.01, compared with the INK-ATTAC+MCAO group.

To determine the neuroprotective effects of the local elimination of senescent cells in the brain on ischemic brain injury, the lenti-INK-ATTAC and control lenti-GFP viruses were injected stereotaxically 5 days before MCAO, and the mice were then treated with AP20187 (10 mg/kg/day) for 5 consecutive days from 1 day before MCAO. The neurobehavioral assays comprised NSS, total distance of locomotor activity, and rotarod performance, and were conducted 1, 3, 7, 14, and 21 days after MCAO (Fig. 5A). AP20187 treatment substantially improved weight loss and neurological deficits in the mice (Fig. 5B and 5C) and significantly reduced the content of typical SASP factors, namely MMP-3, IL-1α, and IL-6, in the injured brain 7 days post-MCAO in the lenti-INK-ATTAC virus-infected mice (Fig. 5D). The mRNA levels of senescence markers p16^INK4a^ also decreased after AP20187 treatment (Fig. 5E). Local elimination of senescent cells in the brain by using a lenti-INK-ATTAC viral vector thus has neuroprotective effects against acute hypoxic brain injury in mice.

## Discussion

The only medication approved worldwide for treating AIS is rt-PA, but the 3- or 4.5-h time limitation for administration and the notable side effects of hemorrhage and neurotoxicity lower its clinical usage.^1^, ^2^ This study is the first to identify senescent astrocytes and CECs in the ipsilateral brain of mice with AIS and demonstrate the change in brain activity during senolytic therapy. In addition, the use of a novel lenti-INK-ATTAC viral vector highlighted the effects of acute brain senescence on hypoxic brain injury in mice that underwent MCAO.

Cellular senescence was first discovered in 1961 when human embryonic fibroblasts undergoing serial subcultures revealed a low capacity of replication but remained alive.^9^ Senescent cells are characterized by several properties. The foci of DNA damage, especially telomeres, are often detected in senescent cells with increased β-galactosidase activity. SASP is a phenotype associated with senescent cells and releases a number of proinflammatory and fibrolytic cytokines, growth factors, proteases, and immune modulators, including IL-1, IL-6, and IL-8.^30^ Cellular senescence has negative repercussions, many of which are caused by SASP, 31 and is accelerated by oxidative stress-mediated mitochondrial malfunction, which disrupts cellular signaling cascades.^32^ Senescent cells accumulate because of aging and at the pathological sites of various diseases, including critical diseases that cause morbidity, mortality, or high healthcare cost.^7^ Neuronal cells of the central nervous system (CNS) include neurons, astrocytes, microglia, oligodendrocytes, and neural stem cells, all of which demonstrate senescent properties of various diseases.^33^-^35^

Cellular senescence in neuronal cells contributes to the pathogenesis of several neurodegenerative disorders through (1) neuroinflammation, which not only leads to senescence but also exacerbates the neuronal damage to the neighboring cells;^30^ (2) the feedback mechanism among senescence, mitochondrial dysfunction, and oxidative stress, which is critical in neurodegenerative disorders;^32^ and (3) permanent cell cycle arrest and other blockages of cellular senescence, which reduce the functionality of CNS cells.^36^ Acute senescence is a component of the healing process after tissue damage. Chronic senescence, which is stimulated by continued exposure to stress, induces cell and tissue damage.^13^ One study by Torres-Querol revealed acute senescence in mice with AIS after MCAO.^37^ The study also indicated an increase of p16^INK4a^ in the cytoplasm of neurons and microglia in transient MCAO mice by using an immunohistochemistry staining assay.^37^ Oxidative stress contributes to *in vitro* neuronal cell death caused by OGD and reoxygenation.^38^ When coupled with reoxygenation, superoxide and other reactive oxygen species form and create oxidative stress, which is closely associated with cellular senescence.^39^ In the present study, we calculated the percentage of senescent cells in brain cells, namely neurons, astrocytes, microglia, and CECs, through flowcytometric analysis of mice after acute ischemic brain injury. We determined that although MCAO-induced cellular senescence appears in all brain cells, only astrocytes and CECs demonstrate a significant increase in senescence. The results of flowcytometric analysis and immunohistochemistry staining (Figure S1) revealed a lower expression of the senescence marker p16^INK4a^ in both neurons and microglias after MCAO surgery. Immunohistochemistry staining also revealed the induction of substantial senescence in astrocytes and CECs of the ipsilateral brain of the mice with MCAO and in OGD-stimulated primary astrocytes and CECs. SASP factors were also created by MCAO and OGD in the ipsilateral brain and primary astrocytes and CECs, respectively. In addition, we also calculated the percentage of senescent brain cells in aged mice by using flowcytometric analysis. The percentage of neurons, astrocytes, microglia, and CECs all significantly increased among aged mice compared with among young mice. Therefore, acute senescence, which occurs because of brain pathologies and chronic senescence and is closely associated with aging, may occur in different cell types. Cortical plasticity and recovery after stroke are long-term processes that can last several months among human patients.^40^ Longitudinal tracking is therefore required to record potential pathological changes following stroke.^41^ PET imaging is a strong tool in both research and clinical use because it has been used for metabolic-biochemical-molecular studies *in vivo* and in a minimally invasive manner.^42^, ^43^ Studies using 18F-FDG in both preclinical and clinical settings have consistently reported that 18F-FDG absorption is lower in areas thought to be the site of ischemic injury.^44^ Additionally, experimental studies using monkey and small rodent ischemic stroke models have consistently demonstrated reduced 18F-FDG uptake in areas that include the ischemic core area.^45^ In addition, our study used 18F-FDG PET to observe the damage in mice caused by ischemia. Variation in 18F-FDG binding ratios in each brain region can be used to identify recoverable tissue. Other studies have primarily focused on the short-term change in cell senescence in ischemic brain injury. In the present study, we observed functional and morphological changes up to 21 days after brain injury.

Senolytic therapeutic strategies for neurodegenerative diseases have increasingly garnered attention. Senolytics drugs can ameliorate various brain diseases including acute ischemic brain injury in animal models, but their application in clinical settings entails difficulties.^6^, ^46^ Other methods for removing senescent cells have emerged, including the systemic administration of senolytics such as ABT-263, curcumin, Dasatinib,^24^ Fisetin,^47^ or FOXO4-DRI, a peptide antagonist that disrupts the connection between FOXO4 and p53 and eliminates senescent cells by inducing apoptosis.^48^ Senescence has both positive and negative effects, and the systemic elimination of senescent cells by using senolytic compounds may have deleterious consequences.^49^ The genetic models in which senescent cells were eliminated may provide evidence of the advantages of senolytic therapy and the pathological effects of cellular senescence.^18^, ^50^ In 2012, Baker et al. created a transgenic INK-ATTAC mouse model to remove senescent cells after administering AP20187, which induces dimerization for use in FKBP fusion protein systems.^18^ On this basis, we created an innovative viral vector by rearranging the INK-ATTAC sequence with minor modification. We assessed the neuroprotective effects through the local elimination of senescent cells in the ipsilateral brain of MCAO mice. Our results demonstrate that local senescent cell eradication through the infection of lenti-INK-ATTAC viruses can significantly alleviate neurobehavioral impairment in MCAO mice after AP20187 treatment. By providing a longitudinal observation of mice behavior after MCAO, we offer important insights into the potential therapeutic benefits of local senescent cell removal for neurodegenerative diseases. Moreover, our study suggests a potential therapeutic approach of ischemic brain injury that could minimize the systemic toxicity associated with systemic senolytic therapy.

In the current study, we addressed several key issues regarding ischemic brain injury and cellular senescence. First, the hypoxic environment significantly accelerates the cellular senescence of astrocyte and endothelial cells in ischemic brain injury, and the universal senolytic medication improves AIS-impaired brain activity. Second, the morphological and behavioral changes in mice with ischemic brain injury significantly improved after we used our local senolytic lenti-INK-ATTAC viral vector to eliminate senescent cells in the ipsilateral brain. These results indicate the pathological role of neuronal senescence in AIS and a potential therapy for AIS, the local clearance of senescent brain cells.

## Supplementary Material

Supplementary methods and figure.

## Figures and Tables

**Figure 6:**
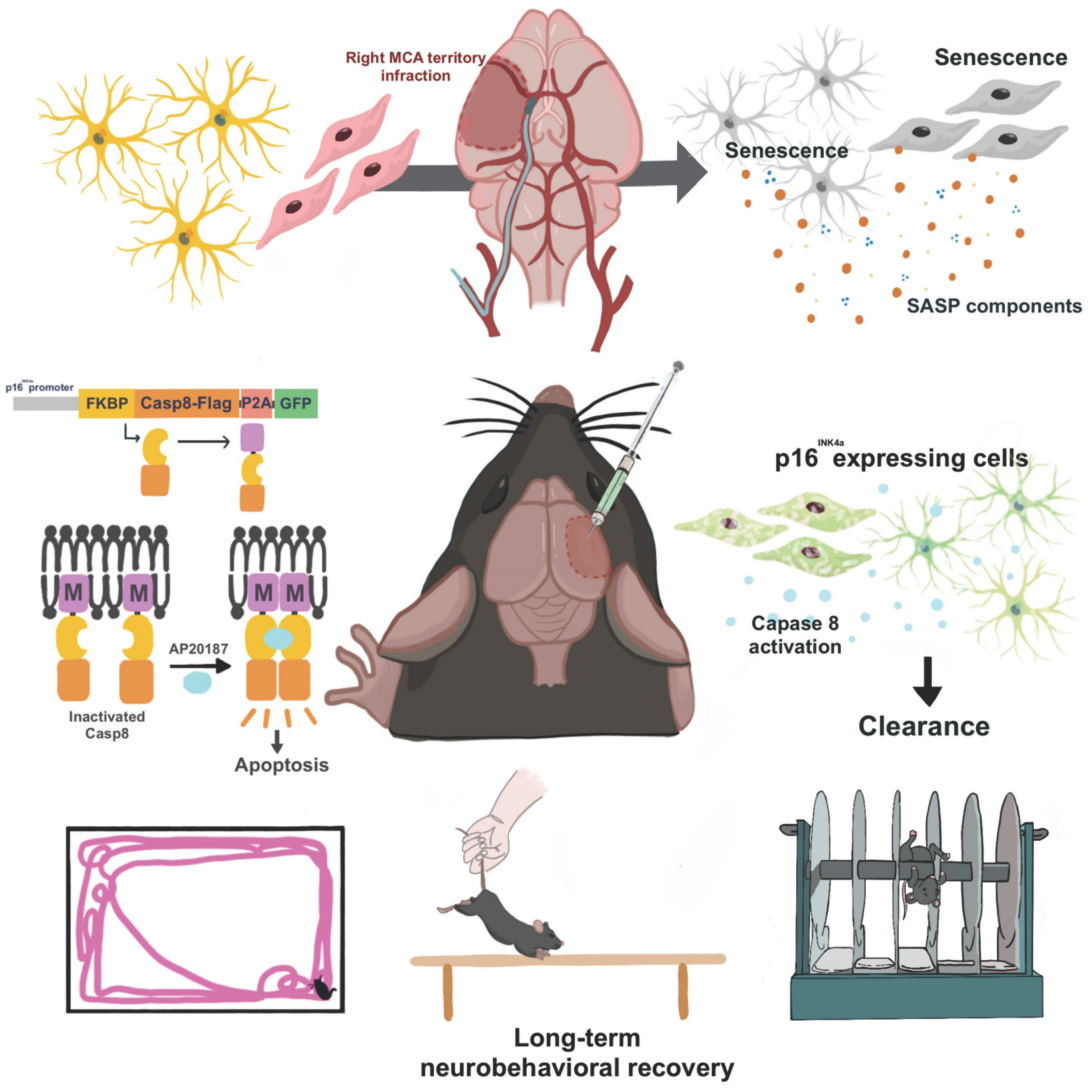
** Local Elimination of Senescent Cells in Ipsilateral Hemisphere to Reduce Ischemic Brain Injury in a Mouse Model of Acute Ischemic Stroke.** MCA: Middle Cerebral Artery; SASP: Senescence Associated Secretory Phenotype

## Abbreviations

AIS: Acute Ischemic Stroke
MCAO: Middle Cerebral Artery Occlusion
CEC: Cerebral endothelial cells
SASP: Senescence Associated Secretory Phenotype
rt-PA: recombinant tissue Plasminogen Activator
NSS: neurological severity score
LMA: Locomotor activity
TTC: 2,3,5-triphenyltetrazolium chloride
FKBP: FK506-binding protein
IL-6: Interleukin 6
CCL8: Chemokine (C-C motif) ligand 8
CXCL2: Chemokine (C-X-C motif) ligand 2
MMP-3: Matrix Netalloproteinase-3
OGD: Oxygen-glucose deprivation
MCA: Middle Cerebral Artery
