# Supplementary material for: Ability of Local Clearance of Senescent Cells in Ipsilateral Hemisphere to Mitigate Acute Ischemic Brain Injury in Mice
Source: Int J Biol Sci. 2023 May 29;19(9):2835–47. doi: 10.7150/ijbs.84060 (PMC10266088; doi:10.7150/ijbs.84060)
Supplement: Supplementary file 1 — Supplementary methods and figure. [file ijbsv19p2835s1.pdf]

Supplemental material

Middle Cerebral Artery Occlusion–Induced Cerebral Ischemia in Mice

We used a mixture of 75% air and 3% isoflurane to anesthetize male C57BL/6 mice, and we kept the mice with 25% oxygen. Rectal temperature was held constant at 37.5 +/- 0.5 °C. According to our previous research, the right middle cerebral artery (MCA) was blocked. A 6-0 monofilament nylon thread (20 mm) coated with silicon (3 mm) was then threaded from the external to the internal carotid artery until the tip obstructed the origin of the MCA[20]. The right common carotid artery was then exposed. After a 30-minute MCA occlusion, the filament was delicately removed during a subsequent brief period of anesthesia (MCAO).The anesthetic was removed once the surgical wound was closed, and the mice regained consciousness.

Neurological Severity Examination

Neurological examination was performed on each mouse immediately before MCAO and at 1 day, 3 days, 7 days, 14 days, and 21 days after injury.[20] An 18-point sliding scale was used to determine the neurological severity scores (NSSs) (normal score, 0; maximum deficit score, 18). The NSS exam consists of balance, reflex, motor, and sensory tests. A score of 1 on the NSS test denotes an inability to complete the test or the absence of a reflex. Consequently, a higher score denotes a more serious injury.

Spontaneous Locomotor Activity and Rotarod Assessments

Before neurobehavioral testing, the mice were trained daily for 3 days to remain on an accelerating rotarod (4–40 rpm over 5 min, with increasing steps of 4 rpm at 30-s intervals) and then underwent MCAO. Before and at 1, 3, 7, 14 and 21 day after MCAO, the mice were placed in an activity monitor (Noldus Information Technology, Wageningen, Netherlands). Locomotor activity (LMA) was recorded automatically by counting the number of beam breaks

during the test. Total beam breaks were recorded in a 15-min period. After the LMA assessment, rotarod performance was evaluated to test balance and coordination by using a Mouse Rotarod (UGO Basile, Varese, Italy). The rotarod rotated from 4 to 40 rpm for 3 min. The time (in seconds) at which each mouse fell from the drum was recorded (for 180 s) using a stopwatch.

Isolation of Cells from Mouse Brain Tissue

The mice were deeply anesthetized and perfused transcardially with 50 mL of ice-cold phosphate buffered saline (PBS). For whole-brain isolation, mice were decapitated, and the brains were rinsed using cold PBS. Each brain was cut into small pieces, followed by the addition of a digestion cocktail enzyme mix (50 μL of DNAse I [Thermo-Fisher, Waltham, MA, USA], 1 mg of collagenase type I [Sigma-Aldrich, St. Louis, MO, USA], and 1 mg of dispase (Sigma-Aldrich, St. Louis, MO, USA) added to 10 mL Hanks' buffered salt solution [Gibco, Waltham, MA, USA] for each brain). The sample was filtered twice over a 50-μm nylon filter and centrifuged for 5 min at 300 × *g*. The pellet was resuspended in 10 mL Hanks' buffered salt solution with 2 mM EDTA (Duchefa, Haarlem, Netherland) and 10% heat-inactivated fetal bovine serum (FBS; Gibco, Waltham, MA, USA). The cells were counted and diluted with PBS and 10% FBS and brought to a minimal concentration of 1×10^6 cells/mL.

Flow Cytometric Analysis and Sorting

All samples were sorted by FacsAria (Becton-Dickinson, San Jose, CA, USA) at 45 psi by using a 70-µm nozzle. The sorted cells were collected into 5-mL microfuge tubes. The sample loader and collection tubes were preserved at 4 °C. Subsequently, a mixture containing fluorochrome-conjugated antibodies with Brilliant Violet 421™ anti-mouse CD31 Antibody (Biolegend, San Diego, CA, USA), Brilliant Violet 421 antimouse/human CD11b (Biolegend, San Diego, CA, USA), Alex Fluor 700-antimouse CD90.2 (Biolegend, San Diego, CA, USA), Alex Fluor 647-antimouse GFAP (eBioscience, San Diego, CA, USA), and p16ARF/CDKN2A antibodies (Novus Biologicals, Littleton, CO, USA) was added. To distinguish between living and dead cells, 7-AAD (50 µg/mL; Biolegend, San Diego, CA, USA) was also added and incubated for 5 min before sorting. The samples were washed with 1 mL PBS + 1% FBS and centrifuged for 5 min at 300 × *g*. Finally, cell pellets were resuspended in 1 mL PBS +1% FBS and immediately subjected to flow cytometry. Spectral overlap was calculated using FACSDiva (BD Bioscience, San Jose, CA, USA) and single stained samples. Altogether, between 100,000 and 1,000,000 events were acquired from each sample to set gates prior to cell sorting.

Tissue Sectioning, Staining, and Imaging

The brain slices were prepared and stained using previously mentioned method.[21] Sections were permeabilized with 0.2% Triton X-100 in PBS and then incubated with primary antibodies and 5% normal goat serum in PBS for 18–24 h. Cellular senescence was detected using the anti-CDKN2A/p16INK4a antibody (Abcam, Cambridge, UK) and taken by EVOS FL Cell Imaging System (Thermo-Fisher, Waltham, MA). NeuN (Abcam, Cambridge, UK), GFAP (Abcam, Cambridge, UK), Iba-1 (Wako Pure Chemicals, Osaka, Japan), and CD31/PECAM-1 (Thermo-Fisher, Waltham, MA, USA) were used to identify specific cell

types. For the dendritic morphology analyses of retrovirally labeling newly generated neurons, the brain sections were prepared 7, 14, and 21 days after viral injection. The green fluorescent protein (GFP) expression of individual newborn neurons was imaged using a confocal spectral microscope imaging system (Leica TCS SP5, Hessen, Germany) through a 40 × 1.40 NA oil immersion objective (Mannheim, Germany). The morphological indices, namely length of primary dendrite, number of branches, and total dendrite length of the individual newly generated neurons, were analyzed using the Imaris software (Oxford Instruments, South Windsor, CT, USA)

Enzyme-Linked Immunosorbent Assay

SASP components, namely matrix metalloproteinase 3 (MMP-3), interleukin-1 alpha (IL-1α), and IL-6, were expressed in the injured brain at 24 h after MCAO. A cell medium from enzyme-linked immunosorbent assay (ELISA) kits (Abcam, Cambridge, UK) was used in accordance with the manufacturer's instructions. Colorimetric detection was conducted using a Multiskan microplate spectrophotometer (Thermo-Fisher, Waltham, MA, USA) at a 450-nm wavelength.

Quantitative Real-Time Polymerase Chain Reaction

cDNA was synthesized from tissue-harvested total RNA by using the SuperScript III kit (Invitrogen, Waltham, MA, USA) in accordance with the manufacturer's instructions. All polymerase chain reactions (PCRs) were conducted in a final volume of 10 μL and in triplicate for each cDNA sample by using a Fast SYBR Green master mix (Applied Biosystems, Waltham, MA, USA) on a StepOnePlus Real-Time PCR system (Applied Biosystems, Waltham, MA, USA) with the following primers and probes[22]: p16INK4a: forward (FW) 5'-GAGCAGCATGGAGCCTTC-3'; reverse (RV) 5'-CGTAACTATTCGGTGCGTTG-3', IL-6: FW 5'-GCCCAGCTGAACTCCTTCT-3'; RV 5'-GAAGGCAGCAGGCAACAC-3', C-C

motif chemokine ligand 8 (CCL8): FW 5'-CGGGTGCTGAAAAGCTACGA-3'; RV 5'-TTGGTCTGGAAAACCACAGCTT-3’, C-X-C motif chemokine ligand 2 (CXCL2): FW 5'-CCCAGACAGAAGTCATAGCCAC-3'; and RV 5'-TGGTTCTTCCGTTGAGGGAC-3'. GAPDH FW 5'-CCCTTAAGAGGGATGCTGCC-3'; RV 5'-TACGGCCAAATCCGTTCACA-3’.

Micro-Positron Emission Tomography Image Acquisition and Processing

Longitudinal micro-positron emission tomography (PET) scans were performed to measure the regional glucose metabolism before and after MCAO, with or without the administration of ABT-263. Each mouse was scanned three times, on Days 1, 3, and 7 after MCAO surgery. The dynamic whole-brain PET images were acquired using a SuperArgus 2R PET scanner (Sedecal). Mice were fasted for 24 h before each scan, and 2-deoxy-2-[18F]-fluoro-D-glucose (FDG; 0.1 mCi/20 g) was injected into the tail vein. After a 30-min uptake period, the animals were anesthetized with 1% isoflurane and transferred to the scanner. The mice were fixed with a customized head holder and anesthetized during scanning with temperature and respiration monitored throughout. All images were automatically coregistered to the magnetic resonance imaging template of the C57BL/6 mice brain, and manual coregistration was performed for minor misalignments in the template. The images were normalized to the mean value of the cerebellum by using the PMOD software, RRID:SCR_016547. To identify the metabolic activity of a specific region of the brain, we used the regional specific binding ratio of the standardized uptake value (SUV) of FDG to the average SUV throughout the cerebellum. SUV is the quantity of incorporated FDG in a specific region of the brain in each animal.

Construction, Production, and Stereotaxic Injection of Engineered Lentiviruses

The lentiviral system was based on an HIV-1-based backbone called pHAGE (plasmid HIV-1 Alex Gustavo George Enhanced; #106281, Addgene, Teddington, UK). The lenti-INK-ATTAC transgenic model (Figure 4A) was synthesized using GenScript (Piscataway, NJ, USA). A 2,617-bp segment of the mouse $p16^{\mathrm{Ink4a}}$ promoter sequence and a FKBP–Casp8 fragment were inserted into the construct[18]. A P2A Nhe1 GFP/STOP codon was inserted 3′ into the ATTAC construct to replace the original IRES-EGFP fragment. The lentiviral particles were generated as previously described using HEK293 cells.[23] The medium was collected 48 h after transfection for the ultracentrifugation to concentrate the recombinant pseudoviral particles. The concentrated lentiviruses were stereotaxically injected at four sites in the cortex and striatum (0.5 μL per site at a speed of 0.25 μL/min) with the following coordinates[23]: 0.4 mm anterior, 2.3 mm lateral relative to bregma, and 1.8 and 3.5 mm below the dura; 2.2 mm anterior, 1.8 mm lateral relative to bregma, and 1.5 mm below the dura; and 2.2 mm anterior, 3.0 mm lateral relative to bregma, and 1.5 mm below the dura.

Supplemental Figure

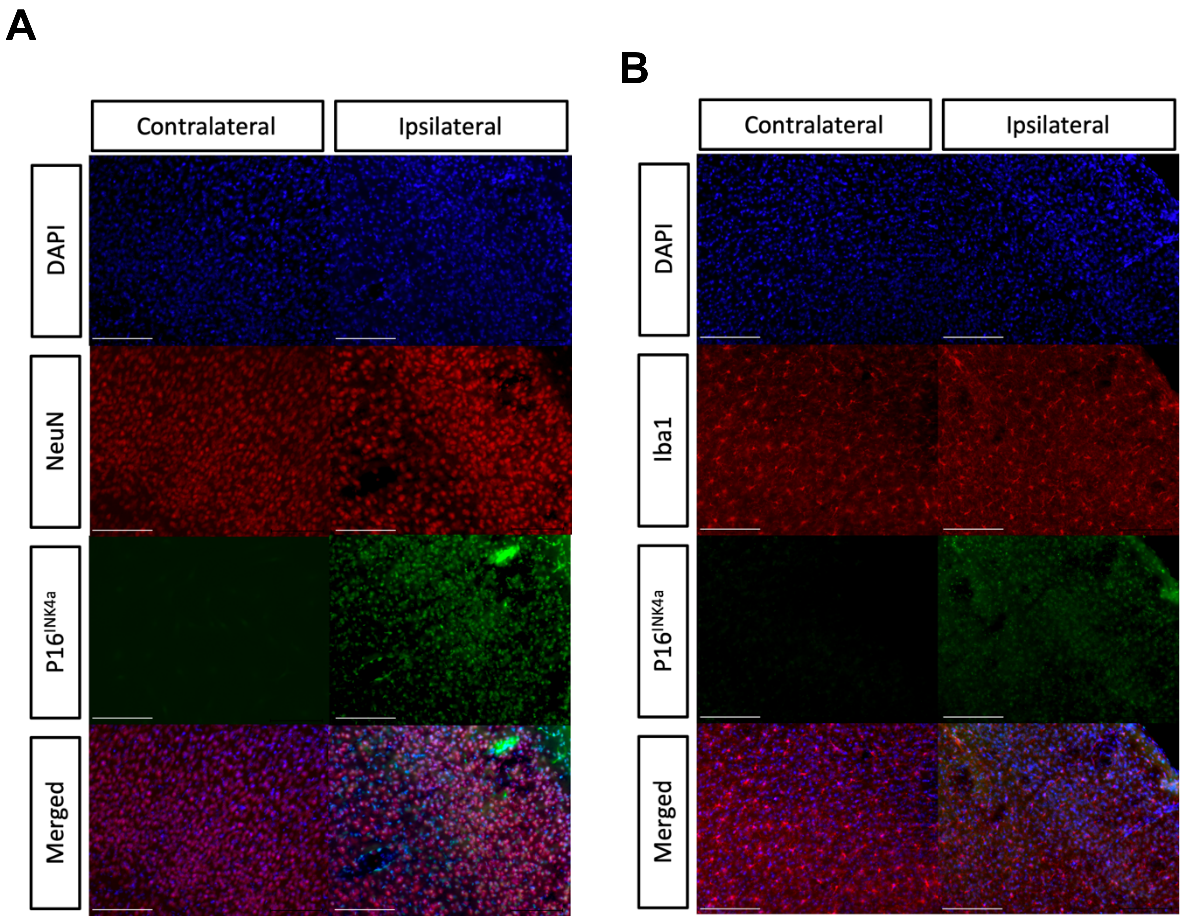

Supplemental. F1

Acute cellular senescence in neurons and microglia of ipsilateral cortex through MCAO

The expression of senescence marker p16^{INK4a} in **(A)** neurons and **(B)** microglias were observed using immunohistochemistry staining and confocal microscopy in the ipsilateral cortex of mice 24 h after MCAO. The white bars represent 125 μm**.**
